# A systematic review of *Neisseria gonorrhoeae* drug resistance development in South Africa

**DOI:** 10.1007/s42770-024-01281-6

**Published:** 2024-04-25

**Authors:** Sinethemba H. Yakobi, Yolisa B. Magibile, Ofentse J. Pooe

**Affiliations:** School of Life Sciences, Biochemistry, University of KwaZulu-Natal, Durban, 4041 South Africa

**Keywords:** Antimicrobial resistance, South Africa, *Neisseria gonorrhoeae*, Azithromycin, Ceftriaxone, Ciprofloxacin, Penicillin, Tetracycline, Spectinomycin

## Abstract

In South Africa, basic healthcare centres treat sexually transmitted infections (STIs) using a syndromic approach. In line with Preferred Reporting Items for Systematic Reviews and Meta-Analyses (PRISMA) recommendations, a complete study of all randomised controlled trials and surveillance data relevant to *N. gonorrhoeae* antibiotic resistance was conducted. To discover papers published between 2002 and 2022, searches were undertaken using PubMed, EMBASE and any other relevant databases. This systematic review extracted a total of 463 articles published between 2002 and 2022 from a variety of online research sources. Seven South African provinces were represented in the studies that were assessed. Mpumalanga and the North West Province did not have any studies that described the identification and monitoring of antimicrobial resistance (AMR). This study presents data obtained from a comprehensive analysis of 2140 isolates, in which we examined the presence of one or more antibiotic resistance. Our findings revealed that out of these samples, 1891 isolates exhibited antimicrobial properties; tetracycline was the antimicrobial resistance that was found the most often (30%), followed by ciprofloxacin (19%) and penicillin (17%). The mean of the isolates was 143, the upper 95% mean was 243, and the standard deviation (SD) was 181.6. All microbiological identification and susceptibility testing processes must be standardised and improved so national organisations can monitor AMR. The nation’s health community must address all identified areas of concern to avoid AMR.

## Introduction

The emergence and spread of antimicrobial resistance (AMR), antimicrobial-resistant genes and antimicrobial-resistant gene determinants have been portrayed as one of the most significant challenges of the twenty-first century as well as a health issue of concern that is rapidly expanding across the globe [1, 2]. An increasing number of microorganisms throughout the world are resistant to various drugs, as has been observed over the past few years [3]. In addition to the direct expense of hospital services, illnesses caused by microbes resistant to antimicrobials result in an economic burden on both the person and society as a whole [4]. According to research conducted only in Europe by the European Centre for Disease Prevention and Control and the European Medicines Agency, the annual cost of AMR to society is estimated to be €1.5 billion [5]. It has been noted that there is a paucity of data that can be accessed to analyse the cost repercussions on a regional or national level when a feasible treatment option for an illness is completely lost as a result of such resistance [6]. The absence of a monitoring system in Africa is a barrier to resolving problems that are associated with the extent of AMR [7]. Several studies conducted in Africa have come to the conclusion that the continent is in need of an effective surveillance system that is capable of collecting data that is exhaustive and sufficient [8–10]. It is therefore imperative that this rising trend be controlled, as bacterial infections pose a heavy challenge to human populations, particularly among children and individuals with immune suppression in developing countries where malnutrition, HIV/AIDS and poor sanitation are prevalent [6, 11]. The existence of AMR isolates and their spread pose a significant risk to public health,these isolates need to be evaluated in relation to clinical situations in the South African context [12]. In South Africa, primary healthcare centres (PHCs) use a syndromic approach to treat sexually transmitted infections (STIs). This ensures that patients receive treatment for the microorganisms that are most likely to be responsible for their infection based on the clinical symptoms they are experiencing [13]. Male urethritis syndrome (MUS) and vaginal discharge syndrome (VDS) account for the majority of STI presentations, according to the distribution of STIs among male and female patients at South African PHC facilities [1]. The lack of optimised and standard laboratory procedures for microbial culture and antibiotic resistance testing has resulted in a loss of specimen collection skills among healthcare personnel [1]. This has also resulted in a scarcity of disease-specific diagnostic protocols for individual STIs and a lack of investment in laboratory infrastructure and research focused on understanding the prevalence of different STIs [14]. It is necessary to do routine aetiological monitoring of STI syndromes in order to keep existing syndromic treatment recommendations up to date and to evaluate their accuracy [13, 15]. Patients who visit sentinel primary health care clinics have been subject to microbiological monitoring by the Center for HIV and Sexually Transmitted Infections (CHIVSTI) at the National Institute for Communicable Diseases (NICD) in Johannesburg since 2006 [13, 16]. According to the findings, *Neisseria gonorrhoeae* is the leading cause of MUS (70–85%), and it is present in 10–20% of patients with symptomatic VDS [16]. The World Health Organization (WHO) kicked off the Global Gonococcal Antimicrobial Surveillance Program (GASP) once again in 2009 with the goals of monitoring the progression of antimicrobial resistance in *Neisseria gonorrhoeae* and gaining a better understanding of how resistance might develop [17, 18]. Ever since this began, the STI laboratory at CHIVSTI has been conducting gonococcal resistance surveys on an annual basis in Johannesburg [13]. In May 2015, the World Health Organization (WHO) created the Global Antimicrobial Resistance Surveillance System and prioritised *Neisseria gonorrhoeae* (*N. gonorrhoeae*) as a pathogen of importance [9]. This was done in order to monitor the spread of antimicrobial resistance around the world. The first two instances of multidrug-resistant *N. gonorrhoeae* infections in Africa were first described in homosexual males; this demographic is regarded as a high-risk group, according to multiple sources [19]. For this reason, it is necessary to have a solid understanding of the drug resistance profile of gonococcal populations that are found in core transmission groups and to include these populations in sentinel surveillance in order to better inform clinical management guidelines and policy design. The purpose of this comprehensive systematic review analysis is to characterise the patterns and trends of antimicrobial resistance shown by *Neisseria gonorrhoeae* in a South African context over the course of the last twenty years, from 2002 to 2022.

## Methods and design

### Methods

**Fig. 1:**
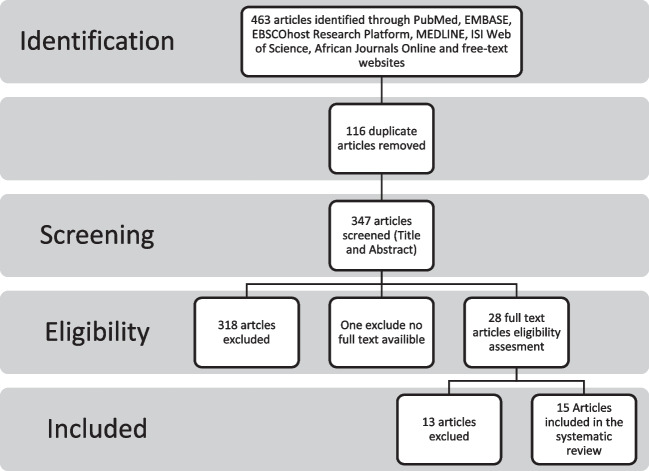
PRISMA Schematic representation of the selection process for papers about antimicrobial resistance in the South African area that were published between May 2002 and May 2022

Screening was performed on the titles and abstracts of all of the articles that were located as a result of the search (see Fig. 1). This systematic review was compiled using the Preferred Reporting Items for Systematic Reviews and Meta-Analyses (PRISMA) guidelines [20].

### Search strategy

The following databases were searched: PubMed, EMBASE, EBSCOhost Research Platform, MEDLINE, ISI Web of Science, African Journals Online and free-text websites. Web searches were conducted using Google Scholar to look for publications written in English that were published between May 2002 and May 2022. It was determined to search the reference lists of pertinent papers for other titles that may be included in the review. The following are some of the key phrases that were used throughout the search: ‘Antimicrobial Resistance’, ‘Antimicrobial Susceptibility’, ‘Surveillance’, ‘Diagnostic’, ‘*N. gonorrhoeae*’ and ‘South Africa’.

### Selection criteria

The articles that were considered for inclusion were those that reported the prevalence of AMR, the availability of AMR surveillance systems or the diagnostic requirements of antibiotic resistance throughout South Africa. Based on the abstract, papers of any kind that included any data at all on the aetiology of the infection and the antibiotic susceptibility pattern were included for further screening. Studies were selected for inclusion or disqualification based on a set of predetermined criteria.

#### Inclusion

The inclusion criteria are as follows: South African reports on the occurrence of AMR in people, both the abstracts and the entire texts in English; testing for drug susceptibility carried out in a controlled laboratory environment; and using predetermined cut-offs for drug susceptibility testing. In population-based investigations, the denominator is the total number of isolates that have been thoroughly characterised. Quantitative studies include those that are experimental, quasi-experimental, prospective and retrospective cohort, case–control and cross-sectional. Observational studies include those that are case series, individual case reports and descriptive cross-sectional studies. Other types of studies include qualitative studies, mixed-methods studies and narrative reviews. In this scoping review, we also take into consideration both systematic studies and opinion publications.

#### Exclusion

The exclusion criteria are as follows: Reports that were published prior to the year May 2002 and before May 2022. Studies solely focused on either *N. gonorrhoeae* but did not include any information on AMR. Studies that do not provide information on the overall number of isolates analysed. Studies were not considered if they have not been carried out in South Africa or if they are only literature reviews that describe the notion of AMR but do not include any interventions. Articles were also excluded if the full text of the article was not accessible.

### Data extraction

The data extraction process makes use of a database that has already been planned, constructed and tested; it was made specifically for the purposes of this study using Microsoft Excel 2013. Article information (first author, year of publication, duration of data collection, country, province and town), study design information (sample size, age group, number of specimens collected and clinical syndrome), pathogen identification and antimicrobial susceptibility testing methodology and antibacterial resistance data were among the types of information that were extracted.

Using a form that was piloted for data extraction and was modified from the JBI data collection tool, two authors (SY and OP) independently extracted the data from each research that is included in the review. Any discrepancies in the data retrieved were settled by the two writers via conversation and reaching an agreement on their findings. During the process of collecting data from each of the included papers, the preliminary version of the data extraction tool was amended as required.

### Data analysis

To derive a standard measurement from the data that were gathered, we computed the prevalence, mean resistance (MR) and inter-quartile range (IQR) of antibiotic resistance for each antibiotic combination. Because there is such a huge degree of variation in the AMR approach, the strength of the relationship between the isolates and the antibiotic-resistant properties was analysed using bivariate analysis. The proportion of the variance for a dependent variable or variables in a regression model was measured using RSquare, and with the use of the *F*-test, we compared the *p*-value for the *F*-test to the level of statistical significance. If the *p*-value was lower than the significance threshold, then the sample data presented sufficient evidence to infer that the regression model fits the data better than the model with no independent variables. This conclusion may be drawn from the fact that the regression model fits the data better.

### Outcomes

To get an insight into the current situation of *N. gonorrhoeae* AMR and to identify information gaps in South Africa.

To offer a summary of the laboratory processes that are currently being used and to place a focus on the needs for diagnostics.

To review available surveillance systems for *N. gonorrhoeae* AMR, focusing on describing the characteristics of these systems.

## Results

This systematic review for the period (2002–2022) extracted a total of 463 articles from a variety of online research sources (Fig. 1). It was decided to eliminate 116 duplicate articles from each of the seven databases. After reviewing the titles and abstracts of the remaining papers, 318 of the 347 items were eliminated, leaving just 29 articles. The criteria for eligibility led to the exclusion of thirteen articles. The exclusions were based on the following factors: the type of article, whether or not it was published within the specified time period, whether or not it reported on the South African population, the absence of antibiotic resistance specifications and results, the fact that some articles solely focused on either *N. gonorrhoeae* but did not include any information on AMR, and the fact that other studies did not provide information on the overall number of isolates that were although one article fulfilled the screening requirements (both the abstract and the title), it was not included in the analysis because its full text could not be obtained. The qualitative systematic review that was conducted included the 15 papers that were left over. Seven South African provinces were represented in the studies that were assessed. Mpumalanga and the North West Province did not have any studies that described the identification and monitoring of AMR that we could find. The province of KwaZulu Natal accounted for 43% of the participants (1749), followed by the province of Gauteng with 31% (1269) of the participants. The Western Cape had a total of 237 participants, accounting for 6% of the total, while the Eastern Cape had 130 participants, accounting for 3% of the total. The last three provinces, Limpopo, the Northern Cape and the Free State, each accounted for just one per cent of the total patient involvement. Twenty percent of the papers, or three of them, had data on AMR for two or more provinces.

The real-time PCR assay, the Etest, the agar diffusion technique and whole genome sequencing were the primary approaches that were used in the antimicrobial susceptibility testing that was carried out (whole genome sequencing). Following the PCR method as the method of choice in 80% (*n* = 12) of the studies was the Etest method, which was used in 60% (*n* = 9) of the studies, followed by the agar diffusion method, which was used in 33% (*n* = 5) of the studies, and finally whole genome sequencing, which was used in 13% (*n* = 2) of the studies. This study presents data obtained from a comprehensive analysis of 2140 isolates, in which we examined the presence of one or more antibiotic resistance. Our findings revealed that out of these samples, 1891 isolates exhibited antimicrobial properties.

We found that the mean of the isolates from the total isolate number of all the studies used in this review combined was 143, the upper 95% mean was 243, the lower 95% mean was 42 and the standard deviation (SD) was 181.6. These results were obtained by doing statistical summaries of the distributions, which summarise a data distribution by presenting the average value of a variable, 95% confidence intervals for each antibiotic resistance, and a measure of how distributed the data is in regard to the mean. We observed a male mean of 221 participants, with an upper 95% mean of 395, a lower 95% mean of 48 and an SD of 286.9. This is in contrast to the female mean of 81 participants, which had an upper 95% mean of 207 and an SD of 208.1. This discrepancy can be attributed to the fact that the majority of the identified articles focused on male participants. On the other hand, the mean number of participants throughout the total population that was recruited was 269, with an upper 95% mean of 472, a lower 95% mean of 66 and a standard deviation of 366.2.

**Fig. 2:**
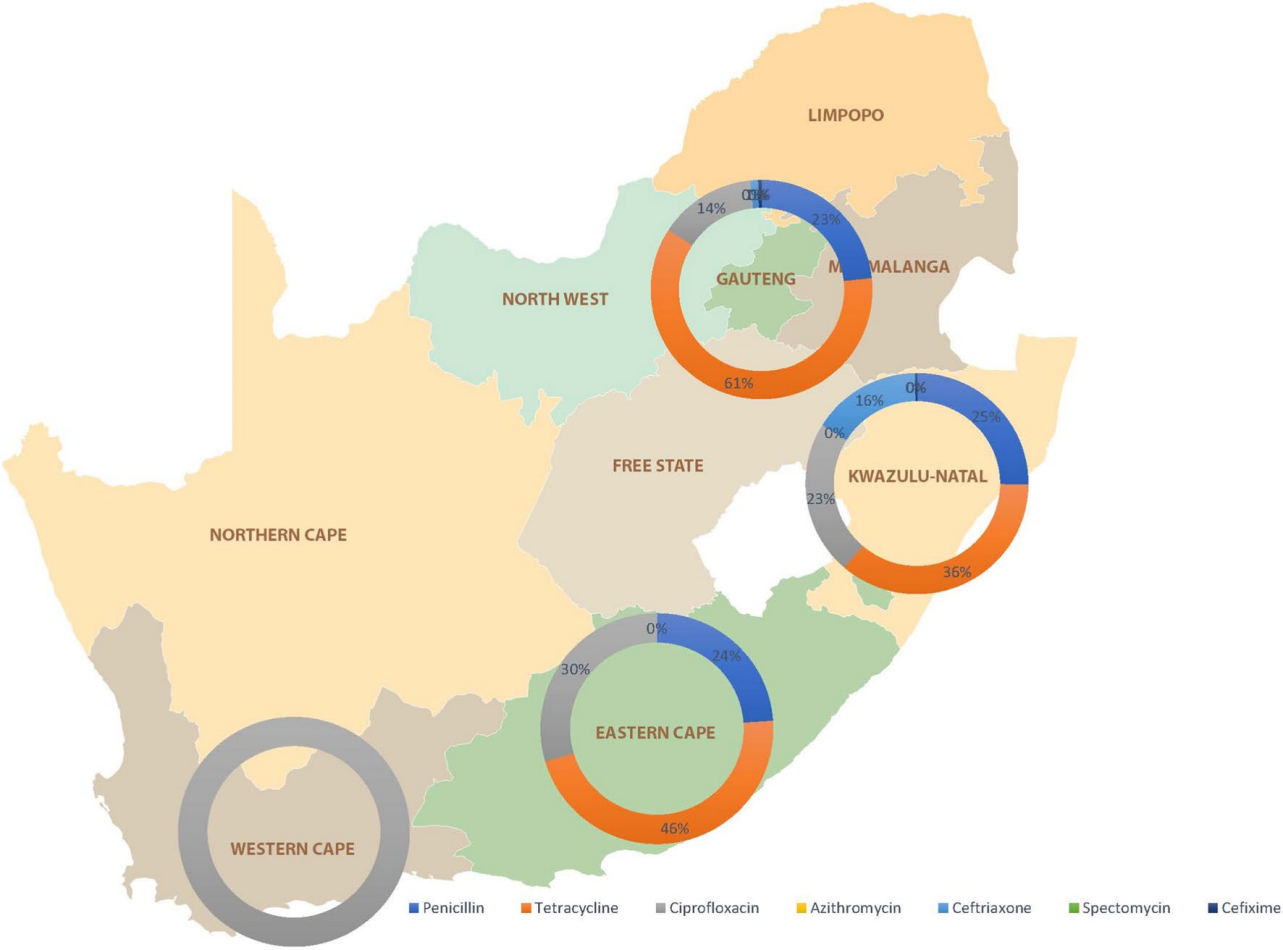
Distribution of identified genotypic *N. gonorrhoeae* AMR reported by four of provinces in South Africa between 2002 and 2022 from studies that provided information on the overall number of isolates and included in this review

The studies that were analysed were from seven different provinces in South Africa; however, AMR was only reported by four of those provinces. There were seventeen instances (24%) of penicillin resistance, thirty-three (46%) cases of tetracycline resistance, twenty-one (30%) cases of ciprofloxacin male participants’ statistical summary resistance and eleven (15%) cases of spectomycin resistance that were attributed to the Eastern Cape Province. One hundred per cent (*n* = 131) of the resistant strains found in the Western Cape Province were resistant to ciprofloxacin. This was the total number of resistant strains found in the province. It was discovered that there were 105 penicillin-resistant (23%) isolates in the province of Gauteng, as well as 274 tetracycline-resistant (61%) isolates, 46 ciprofloxacin-resistant (14%) isolates, 5 azithromycin-resistant isolates (1%) and 3 cefixime-resistant (0.6%) isolates. The KwaZulu-Natal Province was the one that had the highest number of resistant isolates found; this province included 322 penicillin-resistant (25%) isolates, 495 tetracycline-resistant (36%) isolates, 309 ciprofloxacin-resistant (23%) isolates, 217 azithromycin-resistant (17%) isolates and 2 cefixime-resistant (0.15%) isolates. Tetracycline was the antimicrobial resistance that was found the most often (*n* = 802; 30%), followed by ciprofloxacin (*n* = 507; 19%) and penicillin (*n* = 444; 17%), as illustrated in Fig. 2.

Table 1 presents information that was gleaned from each of the full-text publications and is relevant to the topic. In addition, in order to guarantee the data’s accuracy, a second person did their own separate extraction of the same information. When there was a discrepancy, a third author (YM) was given the full-text publications to extract the required data seen in Table 1. The following relevant data were extracted from the study: first author, month and year of publication, duration of study, province and town, number of isolates reported, number of male and female participants, total participant size, anatomical site sampled, age group recruited in the study, clinical symptoms presented by participants, antimicrobial susceptibility testing method and all target antibiotics (penicillin, tetracycline, ciprofloxacin, ceftriaxone, azithromycin, spectomycin and cefixime). The results that were obtained included the strains of bacteria that were isolated, the number of isolates that were tested for antibiotic resistance, the specific antibiotics that were tested for resistance, the percentage of bacteria that were resistant and the genetic epidemiology of ARGs that were found.

**Table 1:** Characteristics of eligible articles

First author	Month and year of publication	Duration	Province	Town	Number of isolates	Males	Females	Total participant size	Anatomical site	Age group	Clinical symptoms	Antimicrobial susceptibility testing method	Penicillin resistance	Ciprofloxacin resistance	Ceftriaxone resistance	Azithromycin resistance	Spectinomycin resistance	Cefixime resistance
Liteboho D Maduna	Nov 20	March 2018–April 2019	Gauteng	Johannesburg	27	42	0	42	Urogenital	22–38	MUS	Microscopy, culture, Etest, molecular detection, DNA preparation and whole genome sequencing	33%	78%	0%	15%	0%	0%
Liteboho D Maduna	Oct 21	Aug 2018–Aug 2019	Gauteng	Pretoria	21	19	2	21	Urogenital	n/a	MUS or VDS	Microscopy, culture, Etest, multiantigen sequence typing (NG-MAST)	86%	62%	Not done	0%	0%	Not done
Nireshni Mitchev	Oct 21	2013–2016	KwaZulu-Natal	Durban	61	31	30	61	Urogenital	15–44	MUS or VDS	Whole genome sequencing (WGS), antimicrobial resistance (NG-STAR)	41%	52%	0%	0%	0%	0%
Santhuri Rambaran	Apr 19	Sept 2013–Dec 2014	KwaZulu-Natal	Pietermaritzburg, Durban	319	506	714	1220	Urogenital	18–42	MUS or VDS	Agar dilution method and polymerase chain reaction with gel electrophoresis	61%	70%	0%	68%	Not done	0.6%
Samuel A. Fayemiwo	Apr 2011	2008	Gauteng	Johannesburg	209	209	0	209	Urogenital	n/a	MUS	Duplex PCR assay	26%	Not done	Not done	Not done	Not done	Not done
Ranmini S. Kularatne	Oct 21	2017–2019	Gauteng, Western Cape, Free State, Eastern Cape, Northern Cape and Limpopo	n/a	685	1000	0	1000	Urogenital	> 18	MUS	Etest, real-time multiplex PCR assay	Not done	Not done	0%	0%	Not done	0.15%
Nireshni Mitchev	Jan 22	June 2015–Jan 2017	KwaZulu Natal	Durban	22	-	-	22	Urogenital	18–60	MUS	Etest, real-time multiplex PCR assay	100%	82%	0%	0%	0%	0%
David A. Lewis	Feb 13	May–July 2012	Gauteng	Johannesburg	2	2	0	2	Urogenital	51,25	MUS	Etest, real-time multiplex PCR assay and agar diffusion	100%	100%	0	50%	0	100%
M De Jongh	Dec 07	2004–2005	Gauteng	Pretoria	141	141	0	141	Urogenital		MUS	Etest, agar dilution methods and NG-MAST	Not done	13%	Not done	Not done	Not done	Not done
Mari De Jongh	Oct 07	March 2004–April 2005	Gauteng	Pretoria	141	366	0	366	Urogenital		MUS	Disc diffusion, Etest and agar dilution	16%	7%	0%	Not done	0%	0%
Prashini Moodley	Jun 06	Nov 03	KwaZulu Natal	Durban	139	139	0	139	Urogenital		MUS	Multiantigen sequence typing (NG-MAST)	57%	22%	0%	Not done	0%	Not done
Ranmini Kularatne	May 22	Jan 2019–Dec 2020	n/a	n/a	542	769	0	769	Urogenital		MUS	Agar dilution methods and Etest	Not done	Not done	0%	0%	Not done	Not done
M.P. Magooa	Feb 13	2006–2007	Western Cape, Gauteng	Cape Town, Johannesburg	40	40	0	40	Urogenital	> 18	MUS	Etest, real-time multiplex PCR assay	Not done	68%	Not done	Not done	Not done	Not done
David A. Lewis	Oct 08	Jan–April 2007	Western Cape, Gauteng	Cape Town, Johannesburg	292	387	0	387	Urogenital, pharygeal, rectal	> 18	MUS	eRNA-based Aptima Combo 2 assay	Not done	36%	Not done	Not done	Not done	Not done
S Govender	Mar 06	2003–2004	Eastern Cape	Gqeberha	35			80	Urogenital	16–49	MUS or VDS	Disk diffusion method	49%	60%	Not done	Not done	31%	0%
Glynis Oree	Mar 21	Nov 2018–July 2019	KwaZulu Natal	Durban	6	0	307	307	Urogenital	> 18	VDS	Etest	50%	83%	0%	0%	0%	0%

*MUS*, male urethritis syndrome; *VDS*, vaginal discharge syndrome

The number of isolates in some studies presented in Table 1 is lower than the total participant size. This is because some studies also report about other pathogens, not just *N. gonorrhoeae*.

**Fig. 3:**
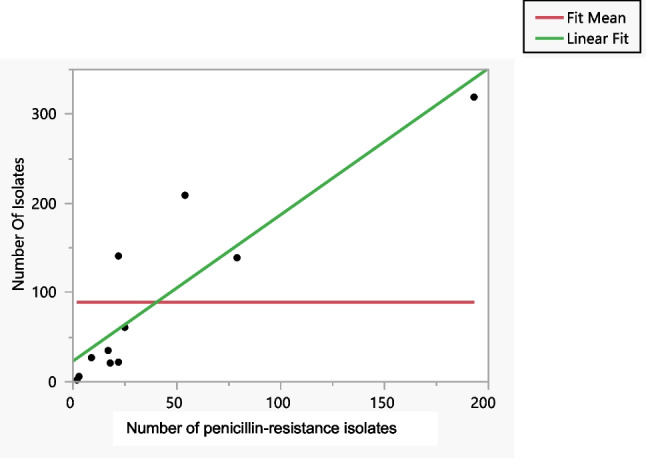
Bivariate fit of the number of isolates by penicillin

**Fig. 4:**
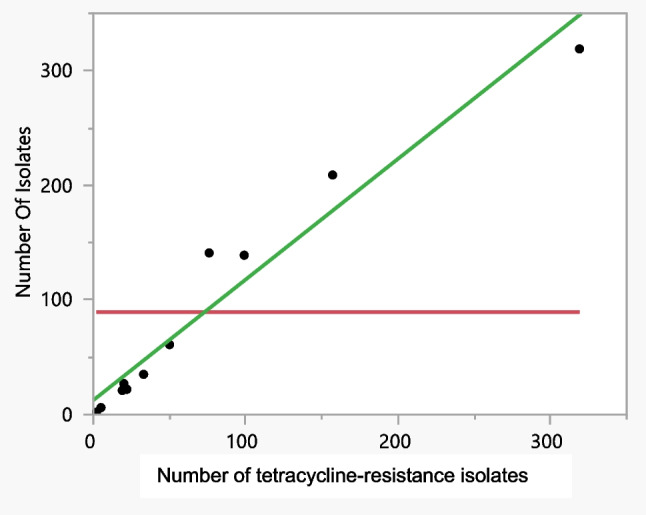
Bivariate fit of the number of isolates by tetracycline

Figures 3 and 4 show an illustration of a simple linear regression, also known as a bivariate regression, which describes the relationship between an explanatory variable and an outcome variable. More specifically, this type of regression is based on the assumption that the explanatory variable influences the outcome variable. The major types of resistance that have been identified are penicillin, tetracycline and ciprofloxacin. We discovered that the more target isolates found, the greater resistance against these three antibiotics was identified. Because no evidence of resistance to ceftriaxone was found in any of the articles that were evaluated, we were unable to establish its effectiveness.

It was discovered that penicillin resistance reflects an RSquare value of 0.8, which corresponds to a mean response of 89.3 over 11 observations, or Sum Wgts. In addition, the ratio of two mean square values, also known as the F-ratio, revealed respective values of 0.5, 0.08 and 1.2 for the antibiotics azithromycin, spectomycin and cefixime, whereas penicillin, tetracycline and ciprofloxacin all exhibited high values (36.2, 160.3, and 25.9, respectively), demonstrating that the AMR mean is far higher than one would anticipate seeing by chance. In addition, the value of the penicillin resistance Prob (f) was found to be 0.0002, which is statistically significant and demonstrates that the independent factors may accurately predict the dependent variable, which in this instance is antibiotic resistance. Tetracycline resistance was discovered to have an RSquare value of 0.9, a mean response of 89.3, a root mean square error of 24.7 in 11 observations or Sum Wgts, and a Prob (f) that was discovered to be 0.0001. All of these results were determined to be statistically significant. Ciprofloxacin resistance was discovered to have an RSquare value of 0.7, a mean response of 95.8, a root mean square error of 60.4 in 13 observations or Sum Wgts, and a Prob (f) that was discovered to be 0.0003. A value of 0.08 for the RSquare statistic, a mean response of 142.8, a root mean square error of 251.1 in 8 observations or Sum Wgts, and a value of 0.49 for the Prob (f) were discovered to be associated with azithromycin resistance. An RSquare value of 0.012, a mean response of 50.4, a root mean square error of 56.9 in 9 observations or Sum Wgts, and a probability of failure of 0.0001 were discovered to be associated with spectinomycin resistance. It was discovered that cefixime resistance reflects an RSquare value of 0.14, a mean response of 144.2, a root mean square error of 223.9 in 9 observations or Sum Wgts, and that the Prob (f) value is 0.31.

## Discussion

In this systematic review, we looked into the evolution of antimicrobial resistance that has taken place in South Africa over the course of the previous twenty years. Since significant funds, human resources and technical expertise are being redirected to other public health priorities, such as HIV/AIDS and tuberculosis, the emergence of multidrug-resistant (MDR) *N. gonorrhoeae* isolates comes at a time when STI control programmes are weak and under-resourced [21, 22]. Antimicrobial-resistant gonococcal strains are now circulating and have been documented in the provinces of the Western Cape, the Eastern Cape, Gauteng and KwaZulu Natal. These microorganisms are primarily distinguished by their combined resistance to oral cephalosporins, quinolones, penicillin and tetracyclines [23]. Even though similar isolates have not yet been reported from any other nations in Africa, history indicates that this may change in the near future [24]. There have not been any instances of XDR genital gonorrhoea that have been verified at this time in terms of either extra ceftriaxone resistance or the combination of resistance to both oral cephalosporins and spectinomycin [25]. The development of extended-spectrum β-lactamases and carbapenems by *N. gonorrhoeae*, which would make all cephalosporins, including ceftriaxone, useless, is the ultimate threat to gonorrhoea control programmes [26]. This threat has already occurred with other Gram-negative bacteria, and despite guidance from the World Health Organization (WHO) that such periodic surveillance should form an integral part of the syndromic management approach, the national department of health in certain regions of South Africa has not conducted any gonococcal antimicrobial susceptibility surveys since the introduction of syndromic management in the late 1990s [27, 28]. In addition, there have been very few studies that have been subjected to peer review and published in the previous twenty years on this subject [29]. In areas where data is available, there are significant variations in testing procedures, such as testing methodologies for the detection of antimicrobial resistance and the kind of media employed for such testing. The sample sizes of some trials are also too small, and none of the investigations used adequate panels of *N. gonorrhoeae* control strains. In addition, there seems to be a lack of validation of potentially relevant resistance results by other laboratories that have shown expertise in conducting antimicrobial susceptibility testing of *N. gonorrhoeae* isolates [30]. This is clearly proven by the assertions of purportedly ceftriaxone-resistant isolates in other countries, despite the fact that it has been established that there are no such strains anywhere in the world [13]. Inadequate storage capacity has also been a problem for several surveys,this has manifested itself in the form of a lack of access to freezers with temperatures of − 70 °C, a failure to use appropriate cryovials and preservative suspensions for gonococci, and a breakdown in the transfer of viable samples from primary testing laboratories to regional reference centres [31].

An increase in the reported prevalence of quinolone-resistant *N. gonorrhoeae* from 0 to 22% over the course of a year was observed during surveillance that was carried out at the University of KwaZulu-Natal [22]. This provided a clear demonstration of how rapidly antimicrobial resistance can emerge. In the absence of continuous microbiological monitoring, the establishment of *N. gonorrhoeae* isolates resistant to antimicrobials may take place undetected and spread quickly [32]. An excellent illustration of this is the Ugandan case, where ciprofloxacin has been used as the first treatment for presumed gonococcal infections for a number of years despite the country’s lack of investment in monitoring programmes [10]. Both males who work in the commercial sex industry (at a rate of 80%) and men who have urethral discharge have been shown to have a high incidence of ciprofloxacin resistance, according to the findings of two recent studies (95%) [10].

Since 2005, the STI Reference Centre at the NICD/NHLS in South Africa has been the organisation in charge of developing and coordinating the National Microbiological Surveillance Programme for STIs [13]. Antimicrobial resistance surveys have been conducted in the provinces of Gauteng (annually since 2007), the Northern Cape (2006), Mpumalanga (2006), the Western Cape (2006–2007), the Free State (2009), the Eastern Cape (2010), the North West Province (2010–2011) and Limpopo (2010–2011) [22]. These studies have shown that gonococci have a high level of resistance to penicillin, tetracycline, and ciprofloxacin. There is an emerging resistance to azithromycin, cefixime and spectinomycin,however, there is no indication that gonococci are resistant to ceftriaxone at this time. More studies and surveillance need to be done all around the country, especially in major townships. The establishment and upkeep of laboratory capacity within these areas will thus be a significant issue in the future.

## Conclusion

Our research concludes with three significant findings: Firstly, South Africa needs more *N. gonorrhoeae* surveillance programmes, as other places currently have none and no data is available. Secondly, there is a significant degree of drug resistance to frequently given antibiotics that has been reported; according to this data, ceftriaxone is the best treatment option for South Africans. Thirdly, the standardisation and quality of microbiological identification and susceptibility testing procedures must be enhanced so that national organisations can monitor the magnitude of the AMR issue. To prevent the public health hazard posed by the development of AMR, the nation’s health community must address all identified areas of concern.

## Data Availability

All data generated or analysed during this study are included in this published article.
